# Enhancing Security in 5G Edge Networks: Predicting Real-Time Zero Trust Attacks Using Machine Learning in SDN Environments

**DOI:** 10.3390/s25061905

**Published:** 2025-03-19

**Authors:** Fiza Ashfaq, Muhammad Wasim, Mumtaz Ali Shah, Abdul Ahad, Ivan Miguel Pires

**Affiliations:** 1 Department of Computer Science, UMT Sialkot Campus, KUST, Sialkot 51040, Pakistan; fiza.muhammadashfaq03@gmail.com (F.A.); muhammad-wasim@skt.umt.edu.pk (M.W.); 2 Department of Computer Science, University of Wah, Wah Cantt 47040, Pakistan; dr.mumtaz.shah@uow.edu.pk; 3 School of Software, Northwestern Polytechnical University, Xi’an 710072, China; 4 Department of Electronics and Communication Engineering, Istanbul Technical University (ITU), Maslak, Istanbul 34469, Turkey; 5 Instituto de Telecomunicações, Escola Superior de Tecnologia e Gestão de Águeda, Universidade de Aveiro, 3810-193 Águeda, Portugal

**Keywords:** cyber security, SDN, machine learning, zero trust, real-time, intrusion detection, intrusion prevention

## Abstract

The Internet has been vulnerable to several attacks as it has expanded, including spoofing, viruses, malicious code attacks, and Distributed Denial of Service (DDoS). The three main types of attacks most frequently reported in the current period are viruses, DoS attacks, and DDoS attacks. Advanced DDoS and DoS attacks are too complex for traditional security solutions, such as intrusion detection systems and firewalls, to detect. The combination of machine learning methods with AI-based machine learning has led to the introduction of several novel attack detection systems. Due to their remarkable performance, machine learning models, in particular, have been essential in identifying DDoS attacks. However, there is a considerable gap in the work on real-time detection of such attacks. This study uses Mininet with the POX Controller to simulate an environment to detect DDoS attacks in real-time settings. The CICDDoS2019 dataset identifies and classifies such attacks in the simulated environment. In addition, a virtual software-defined network (SDN) is used to collect network information from the surrounding area. When an attack occurs, the pre-trained models are used to analyze the traffic and predict the attack in real-time. The performance of the proposed methodology is evaluated based on two metrics: accuracy and detection time. The results reveal that the proposed model achieves an accuracy of 99% within 1 s of the detection time.

## 1. Introduction

**Figure 1:**
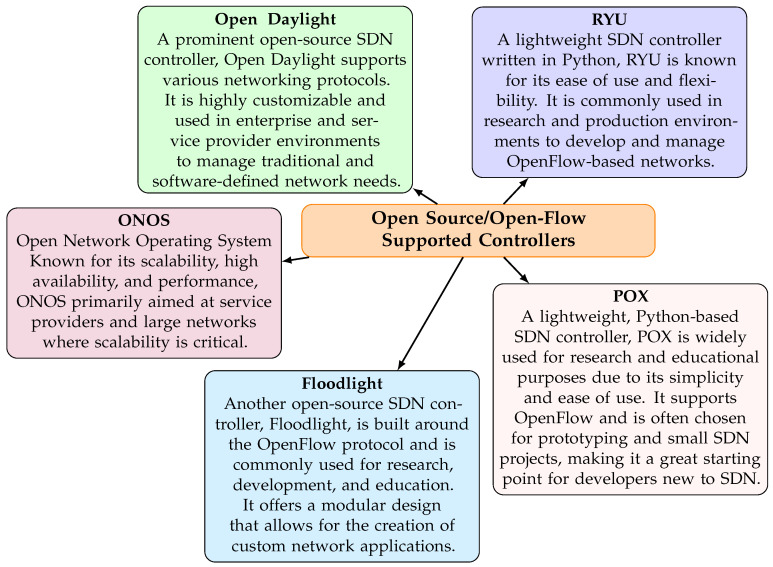
Open source/open-flow supported controllers.

The development of 5G technology has completely changed communication systems. Faster and more reliable connectivity opens up a world of high-capacity applications at the network edge. However, with this paradigm comes a broad attack on the surface, raising security as a significant challenge. Complex networks may now be controlled and protected with the help of a Software-Defined Network (SDN) [1]. SDNs allow for more centralized management and enhance visibility by separating the control plane from the data plane. The researcher [2] makes network administration more responsive and adaptable. SDN has an essential part called the controller, which acts as the brain of the network [3]. Some widely used SDN controllers include RYU, Open Daylight, ONOS, Floodlight, and POX [4], as shown in Figure 1. These include open-source/open-flow-supported controllers. Recently, 5G technology has gained popularity in many industries, but is also open to several threats such as distributed denial-of-service (DDoS) and denial-of-service (DoS) attacks [5]. Every day, on average, 28.7k attacks are launched [6]. According to Neustar’s 2020 Cyber Threats and Trends Report, 151 percent more attacks occurred in June 2020 than in 2019 [6]. In June 2020, the assault volume jumped to 12 GBP from 11 GBP during the same time in 2019.

**Figure 2:**
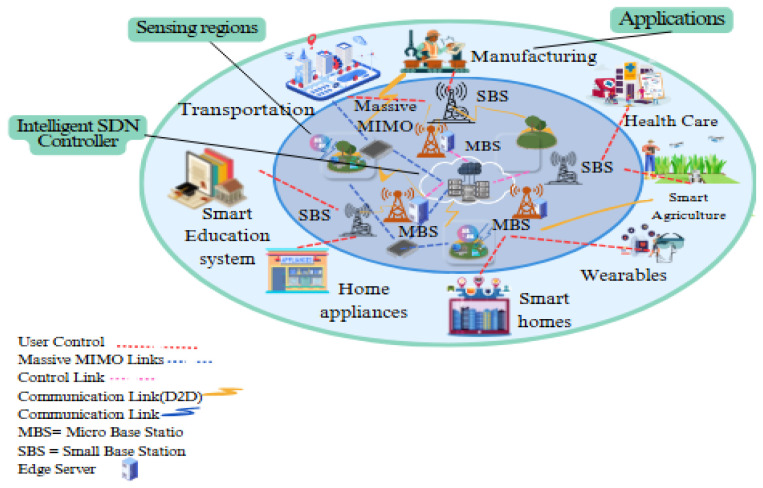
5G network model for intrusion detection model with intelligent SDN.

The transformation of 5G technology is particularly evident in edge computing, where data processing occurs closer to the source, thereby improving efficiency and response times [7]. However, the increasing prevalence of 5G-enabled edge networks also brings new security issues. SDN, a widely accepted revolutionary network, divides the control and data planes to improve network flexibility and management, as shown in Figure 2. The control plane makes forwarding decisions, the data plane handles traffic transmission and forwarding, and the application plane provides clients with programmable and open services [8]. These networks are vulnerable to a wide range of cyber threats due to their highly dynamic and dispersed nature, including DoS and DDoS attacks [9]. The Zero Trust method [10], founded on the principle of never trust, always verify, ensures that all access requests, whether they originate within or outside the network perimeter, are consistently validated and authorized.

Distributed denial-of-service (DDoS) attacks are complex to identify and prevent for traditional security solutions such as firewalls and intrusion detection systems, especially in large-scale networks [11]. Numerous earlier studies used data sets such as UNSW-NB15, NSL-KDD, KDD Cup 99, etc., which do not accurately represent IoT networks’ distinct traffic patterns and vulnerabilities [12]. The lack of publicly available IoT-specific statistics complicates evaluating and validating anomaly detection systems [12]. Detecting DDoS attacks in real-time, essential for minimizing the possibility of harm, is another significant limitation of current approaches. Today, security frameworks, such as intrusion detection systems and firewalls, frequently depend on static settings and predetermined rules. Because of their static nature, they are useless against dynamic and changing attack vectors. The main problem addressed in earlier studies is the lack of effective real-time security mechanisms in SDN environments. The majority of security solutions available are static and cannot be adjusted to the dynamic nature of network environments [12], making them vulnerable to changing security threats.

To address the vulnerability of SDN-enabled 5G networks to cyber-attacks, various machine learning models are applied, including Decision Tree (DT), Logistic Regression (LR), Naive Bias (NB), SVM, KNN, and Random Forest (RF) using the CIDDoS2019 dataset [13]. However, these algorithms do not evaluate for real-time environments. This study addresses this research gap by combining these predictive models with a Mininet simulation environment using a POX controller. The objective is to detect and mitigate real-time attacks. The machine learning-based models dynamically learn and adapt to new cyber threat patterns, improving the controller’s real-time capacity to identify and mitigate network hazards. We build a dynamic and adaptive system that can locate DDoS attacks in SDN by utilizing machine learning within a security and mitigation framework while also forecasting cyberattacks using the Zero Trust concept. The following are the contributions of this paper:

❍ We integrate real-time attack detection in an SDN environment using the POX controller, which has not received as much attention as other SDN controllers such as OpenDaylight and ONOS. We provide the CICDDoS2019 dataset for use in a real-time detection and mitigation framework, in contrast to many research studies that employ out-of-date datasets (for example, NSL-KDD, KDD Cup99), and some studies that use CICDDoS2019 for offline evaluation. In most related activities, real-time attack response and detection time are not crucial assessment criteria. Instead of only evaluating static models, we incorporate attack detection into Mininet, enabling a simulated real-world network environment.
❍ We train six machine learning models on the dataset CICDDOS2019 in a simulation environment using the POX controller separately, and hyperparameter tuning performed with GridSearchCV for the best performance measures.
❍ The proposed framework is extensively tested in a Mininet environment and configurations, demonstrating effective anomaly detection, low resource overhead, and scalability for large-scale Intelligent-SDN networks, including IoT, telematics, WAN, and 5G.

The structure of the rest of this paper is as follows: The literature review is present in Section 2, the methodology is explained in Section 3, Section 4 presents the experimental setup and model development for intrusion detection and mitigation, the results of the simulated environment are in Section 5, the discussions are described in Section 6, and conclusions and future work are presented in Section 7.

## 2. Related Work

The primary purpose of an IDS system is to monitor the computer system for any malicious behavior that might cause any data loss [14]. Software and hardware are used in intrusion detection systems. Machine learning techniques have proven helpful for this purpose since they provide low false alarm rates, high detection rates, and expensive communication costs [15]. This section presents numerous studies on SDN attack detection and mitigation using machine learning. We discuss machine learning-based SDN intrusion detection.

According to M. Latah et al. [16], the majority of researchers examined and developed machine learning methods for intrusion detection, which is the process of detecting any attack that can threaten the network, such as phishing, DoS, and DDOS attacks. Intrusion detection systems (IDS) commonly employ supervised learning, where models using tagged data indicate instances of network traffic as normal conduct or intrusions [17,18]. Supervised learning algorithms learn from similar patterns in deployed data to accurately predict known invasion types. The two primary supervised learning algorithms commonly utilized are the Support Vector Machine and Random Forest [19]. Although Random Forest is used predominantly for regression and classification tasks, SVM excels in classification objectives [20]. SVM demonstrates superior generalization compared to other machine learning methods [20]. However, unsupervised learning learns without labels by automatically classifying the input into many categories. Using unsupervised learning with ML models, a study gleaned relevant information [12]. They used a collection of random variables to represent the data. Class labels are used in unsupervised learning to glean relevant information from input datasets [12].

There are several benefits in connecting commonplace items to the Internet, which can improve our quality of life. However, there are security dangers associated with them also. Mehdi et al. [21] proposed a framework for detecting and mitigating IoT-based smart home intrusions. Their findings demonstrated an accuracy of around 90% in predicting any intrusion detection, and the proposed system could take appropriate action to stop such attempts. Yuan et al. [22] focused on mitigating DDoS damage in SDN networks, where disruptions prevent hosts and the network from efficiently processing signals, preventing user access to normal network services. The centralized structure of SDN makes it more vulnerable to such attacks. A similar approach suggested a two-phase solution: First, they used SVM to establish the optimization parameters [22]. Then, they combined the SVM approach with a genetic cross-validation algorithm to identify the optimization factors and improve traffic flow recognition. Marchese et al. [23] presented a solution addressing the fundamental security challenge confronting SDN systems. More specifically, they substituted a more advanced programmable framework for the traditional architecture but also confronted certain outdated security issues. The SDN strategy presented challenges, because the software-defined networking system is more centralized and vulnerable to hackers.

Similarly, using a machine learning-based technique, Park et al. [24] described a novel method for detecting and preventing network intrusions. In the suggested method, aberrant data was classified to identify SDN infiltration, and preprocessing entailed feature selection. Attack detection and alert generation were adequately handled using the suggested method. The two machine learning classification methods addressed the issues of distinguishing between anomalous and normal data through an audit. G. A. et al. [25] proposed a structure to identify potential abnormalities in SDN networks that used the Network Security Monitor (NSM) technique to detect intrusions without requiring additional host information. Given the flow-based nature of SDN, they opted for an alternative method to detect anomalies within an SDN network. The key goal was to create a system that keeps information open and accessible to both target hosts and possible hackers. To discriminate between different kinds of assaults and legal communications, they used a Random Forest algorithm. They calculated each class’s false positive rate (FPR) and actual positive rate (TPR) before calculating the F1 score to evaluate accuracy. Comparative analysis of key studies and analysis of supervised learning methods for intrusion detection systems are shown in Table 1.

Intrusion detection in software-defined 5G networks relies on artificial intelligence techniques, as outlined by [30], where machine learning algorithms are deployed to recognize potential threats based on flow categorization. Zafari et al. [27] addressed impacts of anticipating hosts in a software-defined network using machine learning techniques. To help the SDN controller anticipate assaults, specific security rules were established. Various machine-learning methods were employed to define these rules. They used the K-means algorithm to classify the data by dividing it into categories known as attack and standard data. Existing IDS are challenging to maintain as they are in confined locations, and it is difficult to identify attacks in such conventional networks intelligently [27]. J. et al. [26] proposed an IDS framework for software-defined 5G networks comprising three distinct layers: the data and intelligence layer, the management and control layer, and the forwarding layer. The forwarding layer monitored and captured network traffic and facilitated packet transmission between OpenFlow (OF) switches. Meanwhile, the control layer collected and analyzed network flows, using the data to block malicious flows as per controller directives. Similarly, a software-defined 5G network implemented and discussed supervised machine learning for intrusion detection [26]. This system, compared to existing methods, reduced the calculation overhead while increasing the accuracy of attack detection. Similarly, Zafari et al. [31], in an SDN network, predicted the impacted hosts using supervised machine learning methods. With the threshold set to 0, the decision tree method produced the most fantastic accuracy of 99.99%. The Bayesian network predicted the assaults with great precision and an average accuracy of 91.68%.

Chao et al. [32] utilized behavior-based SVM classifiers to classify network threats. SVM expedited the learning process while enhancing the accuracy of intrusion detection rates. Similarly, Marchese et al. [14] implemented a strategy reminiscent of SVM’s approach to mitigate malicious threats in traffic flow within SDN networks. The SVM-based model worked effectively, as evidenced by the 80% detection rate of fraudulent activity [14]. When learning unsupervised, methods determine the design based on the unlabeled input data [33]. It examined network traffic data for patterns, abnormalities, or clusters rather than pointing. Unsupervised learning algorithms are valuable for detection because they can identify unknown or innovative forms of incursion. Their ability to detect anomalous patterns in network data allows them to alert users to imminent threats. Table 2 shows a comparison of the proposed method with previous studies.

Saravanan et al. [37] proposed a classification technique for intrusion detection using network security data. The big data tool Apache Spark was used to develop and assess several classification methods, and testing and training times were recorded. However, the authors did evaluate a small number of categorization techniques. They discovered improved outcomes with a good false-positive ratio when compared to the current systems. When evaluating the categorization method in Apache Spark on network security data, better outcomes were obtained compared with current solutions. Decision Tree (DT), Support Vector Machine (SVM), Logistic Regression (LR), and SVM with Stochastic Gradient Descent (SGD) methods have accuracy rates of 96.8%, 93.9%, 92.8%, and 91.1%, respectively. An ML-based model for DDoS attack detection was suggested by Priya et al. [38]. The K-Nearest Neighbors (KNN), Random Forest (RF), and Naive Bayes (NB) classifiers are the three machine learning models that the authors used. Any DDoS assault on the network may be detected using the suggested method. According to the findings of the suggested method, the model has an average accuracy of 98.5% in detecting assaults.

To detect traffic DoS characteristics, Ujjain et al. [39] suggested entropy-based DoS detection, which combines two entropies using CNN and stacked autoencoders (SAE). The CPU use was time-consuming and much more significant. The models’ respective accuracies were 94% and 93%. Gadze et al. [40] suggested deep learning models using Convolutional Neural Networks (CNN) and Long Short-Term Memory (LSTM) to identify and reduce the risk of DDoS assaults that target the centralized controller in Software Defined Networks (SDN). In comparison, the models’ accuracy was lower. The CNN accuracy was 66% and LSTM was 89.63% when the data were split in a 70/30 ratio. DDoS, however, took the longest out of the ten efforts in the LSTM model to identify TCP, UDP, and ICMP. To classify traffic as either DDoS or BENIGN, Ahuja et al. [41] suggested a hybrid machine learning approach called Support Vector Classifier with Random Forest (SVC-RF). After calculating the number of features in the original dataset, the authors produced a new dataset known as the SDN dataset, which contained unique characteristics. According to the findings of the suggested model, the classifier SVC-RF can effectively classify traffic using the SDN dataset with an accuracy of 98.8%. An enhanced deep belief network (DBN) is the foundation of a novel deep learning model presented by Wang et al. [42], which detects network intrusions more effectively. In DBN, they substituted the Kernel-based Extreme Learning Machine (KELM) method for the Back Propagation approach. When compared to current neural network techniques, their suggested model operates more effectively. They assessed several categorization systems and calculated their accuracy. According to the results, the “DBN-KELM” algorithm obtained a 93.5% accuracy rate, while the “DBN-EGWO-KELM” method achieved a 98.60% accuracy rate. Using several machine learning models, Dehkordi et al. [43] suggested a technique to identify DDoS attacks in Software Defined Networks (SDN). The three primary components of the suggested approach were (1) gathering, (2) entropy-based, and (3) categorization. Three distinct datasets were used to test the suggested approach. The findings show that, when applied to an ISCX-SlowDDos2016 dataset, the average accuracy attained by the ML models, i.e., Logistic methods, J48 algorithm, BayesNet algorithm, Random Tree technique, and the  REPTree algorithm was 99.62%, 99.87%, 99.33%, 99.8%, and 99.88%, respectively.

The literature review reveals that most of the proposed methodologies are static and unable to adjust to the dynamic nature of network environments, making them vulnerable to changing security threats [12]. Numerous earlier studies used datasets such as UNSW-NB15, NSL-KDD, KDD Cup 99, etc., which do not accurately represent IoT networks’ distinct traffic patterns and vulnerabilities [12]. The absence of publically available IoT-specific statistics makes it difficult to evaluate and validate anomaly detection solutions [12]. Our study provides real-time network attack detection and mitigation by fusing machine learning approaches with SDN’s dynamic capabilities. We used machine learning techniques to predict attacks in SDN-enabled 5G edge networks, Mininet for simulating networks, and the POX controller for managing SDN.

## 3. Methodology

**Figure 3:**
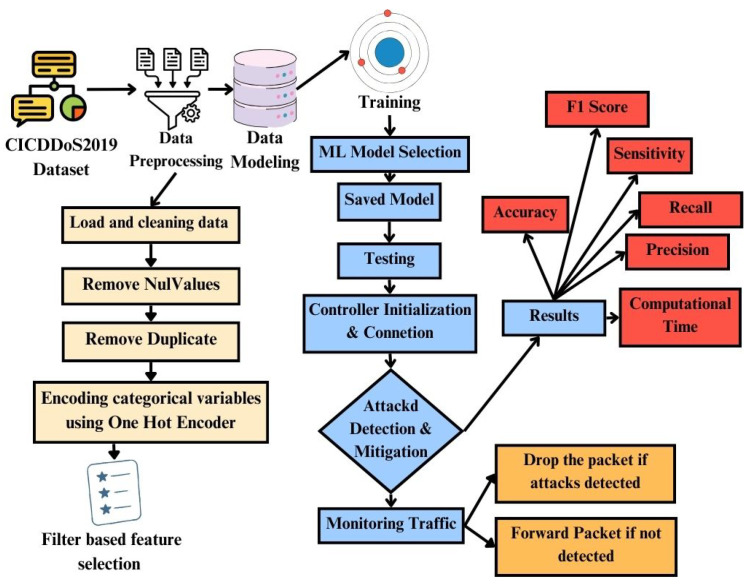
Proposed Methodology for Intelligent SDN.

This section presents a systematic approach to identify and prevent distributed denial of service (DDoS) attacks in a software-defined network with machine learning techniques, depicted in Figure 3. First, we present the benchmark dataset used to train the machine learning models. Secondly, we present data preprocessing techniques, machine learning models, and an evaluation of the results at the end of the section.

### 3.1. CICDDoS2019 Benchmark Dataset

The CICDDoS2019 is a realistic dataset of DDoS attacks and benign traffic [44]. The dataset is publicly available from the Canadian Institute of Cybersecurity’s website (Canadian Institute of Cybersecurity|UNB). Developed by the Canadian Institute of Cybersecurity (CIC) and the Canadian Communications Security Establishment (CSE) https://www.unb.ca/cic/datasets/ddos-2019.html (accessed on 15 January 2025). They used a CIC-Benign-generator to automate the data creation process, which involved instantiating networks of targeted devices using AWS. Log data and network traffic data were recorded and categorized after targeted computers were instrumented and assaulted methodically using a 50-machine attack infrastructure. The dataset’s designers demonstrated a significant effort to improve external validity through their experimental design, target, and attack network architecture, and architectural selection. In addition, there are benign data in this collection. Using CIC-BenignGenerator, benign background traffic is mimicked based on the abstract behavior profiles of twenty-five users. Email, FTP, SSH, HTTP, and HTTPS are the foundations of benign traffic. Previous studies on the CICDDoS2019 dataset, including feature selection methods, real-time attack detection, data pre-processing steps, etc., are shown in Table 3. The data set has 80 features, and Table 4 presents the class distribution of the CICDDoS2019 data set.

The CICDDoS2019 dataset captures two DDoS attack kinds. MSSQL, SSDP, NTP, TFTP, DNS, LDAP, NetBIOS, and SNMP are examples of reflection-based DNS. In this scenario, the real attackers can use the legitimated clients as cover for their attack. It becomes more difficult for victims to distinguish between attackers and users based on the source. DNS, LDAP, NETBIOS, and SNMP are attacks that rely on TCP (MSSQL and SSDP), UDP (NTP and TFTP), or both. Second, some attacks rely on exploits, such as SYN flood, UDP flood, and UDP-Lag. Attackers will send packets to the victim server while spoofing the originating IP address. The victim’s resources will run out as a result of this.

### 3.2. Data Preprocessing

Data pre-processing is a critical step in ensuring the quality and suitability of data for model training. It includes storing categorical variables in a format that machine learning algorithms can understand, normalizing the data to bring all features to a comparable scale, and cleaning the data by dealing with missing values and outliers. In contrast, feature engineering entails generating new features from pre-existing ones to improve the model’s predictive power. The dataset must be accurate, clean, and prepared for usage in machine learning models during the pre-processing stage. After loading the raw data, we eliminated any inconsistent or unnecessary information. We removed missing or insufficient values to maintain the quality of the data. We eliminated duplicate entries to prevent the model’s performance from being incorrect. Using the One Hot Encoding technique, we transformed non-numeric characteristics into a machine-readable format. In addition, we used the filtering technique to choose important characteristics for reducing the dimensionality of the dataset and improving computing efficiency.

### 3.3. Data Modeling

After preprocessing, we trained several machine learning models to determine which is best for DDoS detection. We used 80% of the data for training and 20% for testing to prepare and evaluate machine learning models. We used Decision Tree (DT), Random Forest (RF), Support Vector Machine (SVM), K-nearest neighbors (KNN), Logistic Regression (LR), and Naïve Bayes (NB) for real-time SDN-based intrusion detection because of their classification accuracy, interpretability, computational efficiency, and applicability. RF improves DT’s resilience by lowering overfitting through ensemble learning, whereas DT is selected for real-time detection because of its quick inference time and interpretability. Although its high processing cost restricts its real-time use, SVM is included because of its capacity to handle high-dimensional data and complicated decision boundaries. Although KNN suffers from rising computing costs as the dataset expands, it was chosen for its ease of use and flexibility in responding to novel assault patterns.

Machine learning models are trained to evaluate their accuracy and reliability. The model with the best performance is saved for deployment. The SDN POX controller tracks and controls network traffic and integrates the stored ML model. The machine learning model is implemented in real-time to monitor and analyze network data using Wireshark to detect and mitigate attacks. This stage consists of traffic monitoring, in which incoming network traffic is continuously monitored for irregularities. Traffic is typically sent to its destination if no attack appears. After this, malicious packets are discarded when attacks reduce network interruption. We use sensitivity, recall, accuracy, F1 score, precision, and computation time as performance measures.

### 3.4. Flow of Our Proposed Framework

**Figure 4:**
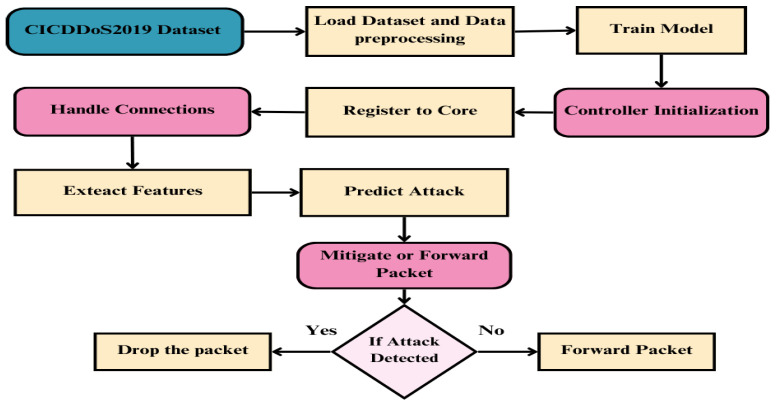
Flow diagram of proposed IntelligentSDN for attack detection and mitigation.

Our proposed framework, IntelligentSDN, uses an organized and structured procedure to identify and stop network assaults. Figure 4 shows the flow diagram that describes the main stages and procedures involved in the system’s operation:

❍ **Initialization:** In this step, we load the dataset for training and building the predictive model. We apply preprocessing, training, and validation procedures used by the model to make sure that it can correctly detect malicious activity. In further phases, the trained model is then saved and made accessible.
❍ **Controller Initialization:** This stage involves the system setting up the POX controller, which is essential for controlling and monitoring the data flow. The controller registers with the core network to create communication channels and ensure it is prepared to handle incoming network traffic.
❍ **Managing Connections:** Managing incoming traffic connections is the focus of this phase. Features are taken out of the packets to offer comprehensive insights into the traffic characteristics. The trained machine learning model processes these characteristics to determine whether the incoming traffic indicates an attack or regular activity.
❍ **Packet Forwarding or Mitigation:** The prediction results determine whether the system sends the packet or mitigates the danger. If packets are found to be malicious, they are discarded to stop the attack from propagating.
❍ **Decision-Making Process:** A decision-making node in the center of the flow chooses what to do with incoming packets. The output of the machine learning model, which acts as the main intelligence for identifying attacks, directs this choice.

Our Intelligent SDN framework, presented in Algorithm 1, identifies and mitigates assaults by combining machine learning techniques with the adaptability of SDN controllers.
**Algorithm 1** Attack detection and mitigation: specific steps and procedures
Our SDN network system uses the following procedures to gather network traffic and categorize it as benign or an attack.
  1:**procedure**  2:**Step 1:** Prepare the dataset for training  3:Load the dataset (CICDDoS2019) containing benign and attack data.  4:Preprocess the dataset:❍Handle missing values.❍Encode categorical features if any.❍Normalize or scale numerical features.  5:Split the dataset into training (80%) and testing (20%) sets.  6:**Step 2:** Define hyperparameter tuning for the Decision Tree  7:Set the parameter grid:❍max_depth = [3, 5, 10, None]❍min_samples_split = [2, 5, 10]❍criterion = [‘gini’, ‘entropy’]  8:Use GridSearchCV to find the best combination of parameters.  9:**Step 3:** Train the Decision Tree model10:Fit the model using the best hyperparameters on the training set.11:**Step 4:** Evaluate the model12:Test the model on the testing set.13:Calculate metrics such as Accuracy, Precision, Recall, F1 Score, and Computation Time.14:**Step 5:** Integrate with Mininet and POX Controller15:Set up a Mininet topology with switches, hosts, and links.16:Assign the POX controller to manage the SDN topology.17:Monitor network traffic using wire-shark.18:Captured real-time traffic analysis.19:**Step 6:** Perform attack detection:20:Use the trained Decision Tree model to classify traffic as “Benign” or “Attack”.21:If an attack is detected:❍Mitigate the attack by blocking the source IP address or limiting bandwidth.❍Update the POX controller rules dynamically to enforce mitigation actions.22:**Step 7:** Display results23:Log detected attacks and mitigated traffic details.24:Visualize the model’s performance metrics and computation time.25:**end procedure**


## 4. Experimental Setup and Model Development

**Figure 5:**
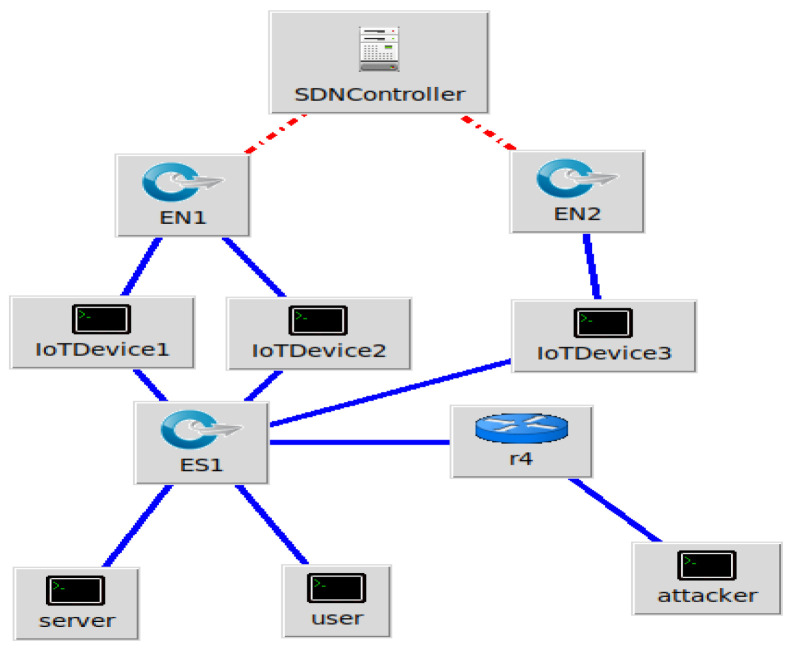
IntelligentSDN security framework.

We use Mininet (Stanford, CA, USA) for implementation to test the effectiveness of machine learning techniques for real-time attack detection and mitigation in SDN. Using the CICDDoS2019 dataset, we integrate machine learning models and Mininet to simulate networks as shown in Figure 5. We explain the threat prevention techniques incorporated into our SDN-based architecture. The SDN controller instantly isolates or blocks the attack by dynamically updating flow rules when it detects a malicious traffic flow. Automated flow table updates are used to do this, blocking questionable IP addresses or unusual traffic patterns to stop further spread. Rate limitation is also used to reduce the danger of distributed denial-of-service (DDoS) attacks by controlling excessive bandwidth from possible attackers. The system uses anomaly-based policy updates for adaptive security, which enables the ML model to improve detection tactics gradually. By ensuring that threats are not only recognized but also actively eliminated, these mitigation techniques enhance network resilience and preserve service continuity. We train and test each well-known machine learning algorithms one at a time using hyper-parameter tuning with GridSearchCV, utilizing 5-fold stratified cross-validation. We used a Windows 10 computer with a 64-bit CPU and 12 GB of RAM and Ubuntu 20.04 as the guest operating system in Oracle’s Virtual Box to set up the experimental environment. We use Mininet and POX controllers on Ubuntu. The POX SDN controller is an open-source controller for OpenFlow/SDN that runs on Python 3.5, and a Mininet that supports OpenFlow 1.3 use in a virtualized environment. We created custom topologies using Python code, while MiniEdit makes it easier to create virtual network topologies. In Table 5, the setup specifications are shown. Mininet investigates numerous networking topologies, and POX SDN provides effective network administration. Actions based on the prediction of the machine learning model are part of the mitigation procedure in the script supplied for the POX controller. The script drops malicious packets to reduce an attack’s impact when detected.

### 4.1. Hyperparameters

We thoroughly investigate several hyperparameters in the process to find the best combination that improves the model’s performance. In this procedure, we use the Grid Search approach, a method included in the Python package Sklearn. The performance of these models is greatly influenced by the number of hyperparameters, such as max depth, min-samples-split, min-samples-leaf, and n-estimators, among others. The Grid Search model performs a cross-validated assessment for every set of hyperparameters. It divides the data into many subsets (folds) and tests the model’s performance on some subsets while training it on others.

For instance, the hyperparameters optimized include n_estimators being varied across values like [50, 100, 200], the maximum depth being set to different sizes [10, 20, None], and min_samples_split representing a minimum of two, five, or ten samples needed to divide an internal node. Similarly, the value ranges for the remaining hyperparameters are set. After the GridSearchCV process, we use the performance metrics on the validation data to determine the optimal set of hyperparameters. In the context of the problem domain under discussion, these optimal hyperparameters reflect the setup that best improves model performance and allows for more precise predictions. The process of finding the optimal values for a model’s parameters that are not discovered immediately from the training data is known as hyperparameter tuning. These factors influence the model’s capacity to generalize and function well on unknown data.

### 4.2. Hyper-Parameter Tuning for Support Vector Machine (SVM)

❍ C: The parameter for regularization.
❍ γ (Gamma): RBF kernel kernel coefficient.
❍ Kernel: sigmoid, poly, rbf, and linear.

The objective function for a Support Vector Machine (SVM) is defined as:

(1) $$\min_{w}\frac{1}{2}{∥w∥}^{2}+C\sum_{i=1}^{n}\xi_{i}$$

Subject to:

(2) $$y_{i}\left(w^{T}x_{i}+b\right)\geq 1-\xi_{i},\;\xi_{i}\geq 0$$

where:

❍ *w*: Weight vector.
❍ *b*: Bias term.
❍ *C*: Regularization parameters controlling the trade-off between maximizing the margin and minimizing the classification error.
❍ $\xi_{i}$: Slack variables to handleincorrect classification.
❍ $y_{i}$: True label of the *i*-th data point ($y_{i}\in \left\{-1,1\right\}$).
❍ $x_{i}$: Feature vector of the *i*-th data point.

#### 4.2.1. Kernel Function

The kernel function maps the data to a higher-dimensional space to make it linearly separable. Standard kernel functions include:

❍ Linear Kernel:
  (3) $$K\left(x,x^{\prime }\right)=x^{T}x^{\prime }$$
❍ Radial Basis Function (RBF) Kernel:
  (4) $$K\left(x,x^{\prime }\right)=\exp(-\gamma ∥x-x^{\prime }{∥}^{2})$$
  Here γ is the kernel coefficient.

#### 4.2.2. GridSearchCV Hyper-Parameter Tuning

GridSearchCV optimizes SVM hyperparameters by searching over a predefined grid. The hyperparameters tuned include:

❍ Regularization parameter (*C*):
  (5) C∈{0.1,1,10,100}
❍ Kernel coefficient (γ) for RBF kernel:
  (6) γ∈{scale,auto,0.01,0.1}
❍ Kernel types: linear, rbf, poly

### 4.3. Hyper-Parameter Tguning for Random Forest (RF)

❍ n_estimators: The forest’s total number of trees.
❍ max_depth: Tree’s maximum depth.
❍ min_samples_split: The minimum number of samples needed to split an internal node
❍ min_samples_leaf: The minimum number of samples needed to be a leaf node.
❍ max_features: The maximum number of features considered for the optimal split.

#### 4.3.1. Gini Impurity

The Gini Impurity is calculated as:

(7) $$G=1-\sum_{k=1}^{K}p_{k}^{2}$$

where:

- $p_{k}$: Proportion of samples of class *k* in a node.
- *K*: Total number of classes.

#### 4.3.2. Information Gain (Entropy-Based)

Information Gain is defined as:

(8) $$IG=H\left(\mathrm{parent}\right)-\sum_{i=1}^{n}\frac{|N_{i}|}{\left|N\right|}H\left(N_{i}\right)$$

where:

❍ *H*: Entropy.
❍ H(parent): Entropy of the parent node.
❍ *N*: Total number of samples in the parent node.
❍ $N_{i}$: Number of samples in the *i*-th child node.
❍ $H(N_{i})$: Entropy of the *i*-th child node.
❍ *n*: Number of child nodes after the split.

#### 4.3.3. Prediction

The prediction of the Random Forest model is based on the majority vote from all trees:

(9) $$\hat{y}=\mathrm{majority}\;\mathrm{vote}\;\mathrm{from}\;\mathrm{all}\;\mathrm{trees}$$

#### 4.3.4. GridSearchCV Hyperparameter Tuning

To optimize the Random Forest model, GridSearchCV searches over a predefined set of hyperparameters:

Number of trees in the forest:

n_estimators∈{10,50,100,200}

Maximum depth of the tree:

max_depth∈{5,10,20,None}

Minimum number of samples required to split an internal node:

min_samples_split∈{2,5,10}

Minimum number of samples required to be at a leaf node:

min_samples_leaf∈{1,2,4}

### 4.4. Hyper-Parameter Tuning for Logistic Regression (LR)

❍ C: The strength of regularization (inverse of the regularization parameter (Γ$γ)
❍ Solver: Optimization algorithm.
❍ Penalty: Elasticnet, L1, L2, or no regularization type.

The objective of logistic regression with regularization is to minimize the following function:

(10) $$\min_{w}\frac{1}{n}\sum_{i=1}^{n}\left[-y_{i}\log\sigma \left(w^{T}x_{i}\right)-\left(1-y_{i}\right)\log\left(1-\sigma \left(w^{T}x_{i}\right)\right)\right]+\frac{\lambda }{2}{∥w∥}_{2}^{2}$$

where:

❍ $\sigma \left(z\right)=\frac{1}{1+e^{-z}}$: Sigmoid activation function.
❍ $\lambda =\frac{1}{C}$: Regularization strength, where *C* is the inverse of λ.
❍ ${∥w∥}_{2}^{2}$: L2 regularization penalty to control overfitting.

#### 4.4.1. Regularization Penalties

Regularization helps prevent overfitting by penalizing the magnitude of model coefficients. Logistic regression supports the following types:

##### L1 Regularization

Encourages sparsity in the coefficients by penalizing the absolute values:

$${∥w∥}_{1}=\sum_{i=1}^{n}\left|w_{i}\right|$$

##### L2 Regularization

Discourages large coefficients by penalizing their squared values:

$${∥w∥}_{2}^{2}=\sum_{i=1}^{n}w_{i}^{2}$$

#### 4.4.2. Hyperparameter Tuning with GridSearchCV

To optimize logistic regression, GridSearchCV is used to search for the best combination of hyperparameters:

❍ Regularization Strength (*C*):
  C∈{0.01,0.1,1,10,100}
❍ Penalty (Regularization Type):
  penalty∈{l1,l2}
❍ Solver: The choice of solver depends on the type of penalty:
  − liblinear: Supports both L1 and L2 penalties.
  − saga: Suitable for large datasets and supports both L1 and L2 penalties.

### 4.5. Hyperparameter Tuning for Decision Tree (DT)

❍ max-depth: maximum tree depth.
❍ min_samples_split: To separate an internal node, the bare minimum of samples is needed.
❍ min_samples_leaf: A leaf node must have a minimum of one sample.
❍ Criteria: Entropy or Gini.

#### 4.5.1. Gini Impurity (Same as RF)

The Gini Impurity is calculated as:

(11) $$G=1-\sum_{k=1}^{K}p_{k}^{2}$$

where:

❍ $p_{k}$: The probability of a data point belonging to class *k*.
❍ *K*: The total number of classes.

#### 4.5.2. Entropy

Entropy is defined as:

(12) $$H=-\sum_{k=1}^{K}p_{k}\log_{2}\left(p_{k}\right)$$

where:

❍ $p_{k}$: The probability of a data point belonging to class *k*.
❍ *K*: The total number of classes.

#### 4.5.3. Information Gain

Information Gain measures the reduction in entropy after splitting the dataset and is defined as:

(13) $$IG=H\left(\mathrm{parent}\right)-\sum_{i=1}^{n}\frac{|N_{i}|}{\left|N\right|}H\left(N_{i}\right)$$

where:

❍ H(parent): Entropy of the parent node.
❍ *N*: Total number of samples in the parent node.
❍ $N_{i}$: Number of samples in the *i*-th child node.
❍ $H(N_{i})$: Entropy of the *i*-th child node.
❍ *n*: Number of child nodes after the split.

#### 4.5.4. GridSearchCV Hyperparameter Tuning

To optimize the decision tree, GridSearchCV searches over a predefined set of hyperparameters:

**Maximum depth of the tree:**

max_depth∈{3,5,10,None}

**Minimum number of samples required to split an internal node:**

min_samples_split∈{2,5,10}

**Minimum number of samples required to be at a leaf node:**

min_samples_leaf∈{1,2,4}

**Criterion for measuring the quality of a split:**

criterion∈{gini,entropy}

### 4.6. Hyper-Parameter Tuning for Naive Bias (NB)

Alpha: For MultinomialNB and ComplementNB, the additive smoothing parameter. There are no important hyperparameters for GaussianNB.

#### 4.6.1. Posterior Probability

Naive Bayes is based on Bayes’ Theorem, which calculates the posterior probability as:

(14) $$P\left(y|x\right)=\frac{P(x|y)P(y)}{P(x)}$$

where:

❍ P(y∣x): Posterior probability of class *y* given data *x*.
❍ P(x∣y): Likelihood of data *x* for class *y*.
❍ P(y): Prior probability of class *y*.
❍ P(x): Evidence, which normalizes the probabilities.

#### 4.6.2. Gaussian Naive Bayes (GaussianNB)

For continuous features, Gaussian Naive Bayes assumes that $P(x_{i}|y)$ follows a normal distribution:

(15) $$P\left(x_{i}|y\right)=\frac{1}{\sqrt{2\pi \sigma_{y}^{2}}}\exp\left(-\frac{{\left(x_{i}-\mu_{y}\right)}^{2}}{2\sigma_{y}^{2}}\right)$$

where:

❍ $\mu_{y}$: Mean of the feature for class *y*.
❍ $\sigma_{y}^{2}$: Variance of the feature for class *y*.

**Multinomial Naive Bayes (MultinomialNB)**

For discrete features, such as text or count data, Multinomial Naive Bayes models $P(x_{i}|y)$ as:

$$P\left(x_{i}|y\right)=\frac{n_{yi}+\alpha }{\sum_{j}\left(n_{yj}+\alpha \right)}$$

where:

❍ $n_{yi}$: Count of feature *i* in class *y*.
❍ $\sum_{j}n_{yj}$: Total count of all features in class *y*.
❍ α: Smoothing parameter to handle zero probabilities.

#### 4.6.3. Hyperparameter Tuning with GridSearchCV

To optimize the Naive Bayes model, the following hyperparameters are tuned using GridSearchCV:

Smoothing Parameter (α)**:**

α∈{0.1,0.5,1.0,2.0}

Model Type:

❍ GaussianNB: For continuous features.
❍ MultinomialNB: For text or count-based features.

The SDN Controller controls the network and guides data flow across the various connected devices, as shown in Figure 5. The controller connects to two essential network components (EN1 and EN2), which act as gateways for traffic control. By connecting these components, IoT devices (IoTDevice1, IoTDevice2, and IoTDevice3) allow for centralized management of IoT activities. The system has a router (R4) to manage additional routing responsibilities and an ES1 switch for increased traffic distribution. The network has critical endpoints, such as a server, a user, and a possible attacker, raising operational and security issues. The logical separation in SDN networks enhances flexibility, scalability, and security, as illustrated by the red and blue connections, which stand for control and data channels, respectively.

**Figure 6:**
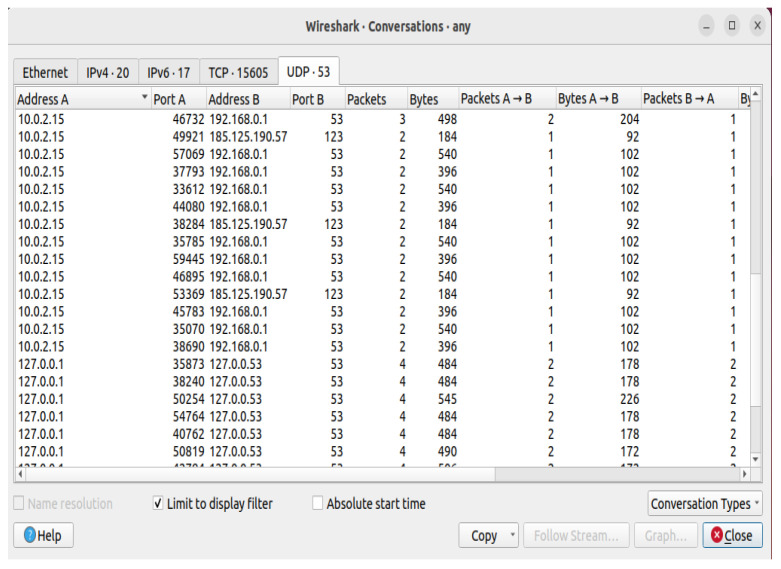
UDP and TCP traffic between hosts in an SDN environment: an analysis of Wireshark conversations.

**Figure 7:**
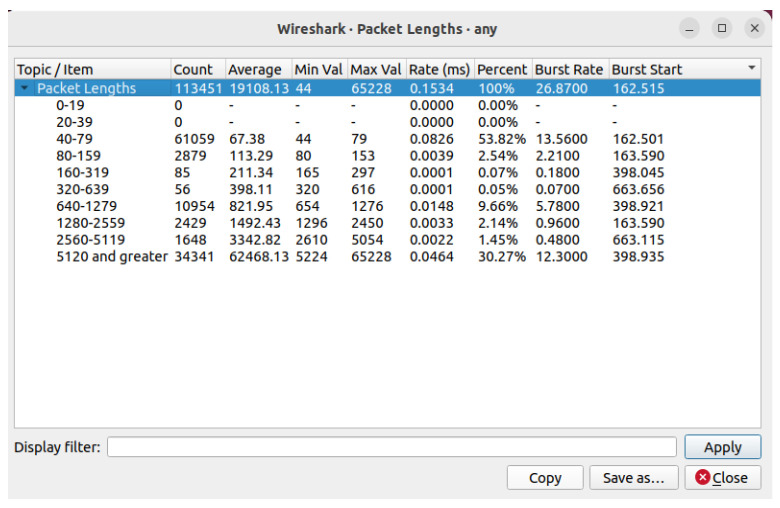
Wireshark Packet length distribution: a statistical study of network traffic recorded.

The POX controller has security components and a Python script that automates the execution of experiments in various network settings. The main tasks include topology development, dataset production, monitoring, and policy changes. The framework is tested and improved by simulating real-world situations in a simulation environment. We enforce micro-segmentation, continuous authentication, and stringent access controls via the SDN controller. Machine Learning (ML) and Zero Trust Security in SDN improve network security by implementing dynamic access control, continuous authentication, and micro-segmentation. By limiting illegal connections between various network zones, micro-segmentation in SDN dynamically separates network segments, preventing attackers from moving laterally. The SDN controller changes the flow tables to isolate compromised devices and stop malicious traffic if an intrusion is found, as shown in Figure 6 and Figure 7. Based on real-time behavioral analysis, continuous authentication, powered by machine learning, ensures continuous identity verification. ML models look at network traffic for abnormalities rather than relying only on login credentials. If found, re-authentication is instantly initiated, or access revocation occurs. With the help of SDN’s centralized control plane, dynamic access control enforces least privilege policies, limiting user and device access to just the required resources. Training machine learning models on network traffic data achieves real-time prediction and mitigation of cyberattacks. We put the network through various attack scenarios to evaluate the efficiency of the framework. With strict security measures and intelligent prediction, the technique guarantees a thorough approach to 5G edge network security.

By preventing malicious traffic, rerouting questionable flows for additional examination, and isolating compromised hosts to limit their access, the SDN controller continuously updates flow tables to counteract assaults. This automatic method minimizes manual involvement and lowers the possibility of a protracted network outage while guaranteeing a prompt reaction to security concerns. To determine if detection accuracy and reaction time stay constant, we test the system under low, medium, and high traffic loads, which are typical of real-world networks that encounter varying traffic situations. This assessment aids in figuring out whether specific machine learning models suffer from high traffic levels, ensuring that the suggested strategy is workable for widespread implementation, including a comparison performance study, detection, etc.

## 5. Results

This section presents the proposed methodology’s performance metrics and results, which are also compared with those of previous methodologies.

### 5.1. Performance Metrics

We use evaluation parameters, including accuracy, precision, recall, F1-score, and computational time, to evaluate each algorithm’s performance. The values of true positives, true negatives, false positives, and false negatives are the foundation of these measures. These performance measures are essential for evaluating our framework and considering detection and mitigation capabilities. Every machine learning method has distinct features for analyzing, making predictions, and learning data points to categorize and identify attacks depending on the tuning parameters. Our proposed model achieves a higher F1-measure, recall, accuracy, and precision than the state-of-the-art models. These indicators thoroughly illustrate our framework’s performance in a range of network environments and threat situations. The performance matrices are defined below:

#### 5.1.1. Accuracy

Evaluates how accurate the attack detection mechanism is overall. It is the percentage of correctly identified cases, including true negatives and true positives, relative to all cases.

(16) $$Accuracy=\frac{\mathrm{True}\;\mathrm{Positives}+\mathrm{True}\;\mathrm{Negatives}}{\mathrm{Total}\;\mathrm{Instances}}$$

#### 5.1.2. Precision

It shows the proportion of attacks that are real threats. Greater accuracy results in fewer false positives.

(17) $$Precision=\frac{\mathrm{True}\;\mathrm{Positives}}{\mathrm{True}\;\mathrm{Positives}+\mathrm{False}\;\mathrm{Positives}}$$

#### 5.1.3. Sensitivity (Recall)

Evaluates the capacity to recognize every actual attack accurately. False negatives decrease when recall is high.

(18) $$Recall=\frac{\mathrm{True}\;\mathrm{Positives}}{\mathrm{True}\;\mathrm{Positives}+\mathrm{False}\;\mathrm{Negatives}}$$

#### 5.1.4. The F1-Score

The harmonic mean of recall and accuracy maintains a balance between the two.

(19) $$F1=\frac{2*Precision*Recall}{\mathrm{Precision}+\mathrm{Recall}}$$

#### 5.1.5. Computational Time

The time it takes for a computer to complete a calculation or activity is known as computational time and referred to as execution time or running time.

(20) CT=EndTime−StartTime

### 5.2. Performance of Machine Learning Classifiers

**Figure 8:**
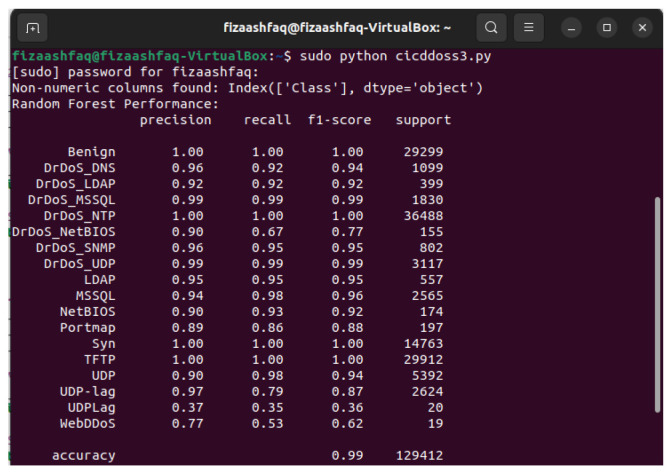
Random Forest performance metric evaluation on the Mininet SDN network.

**Figure 9:**
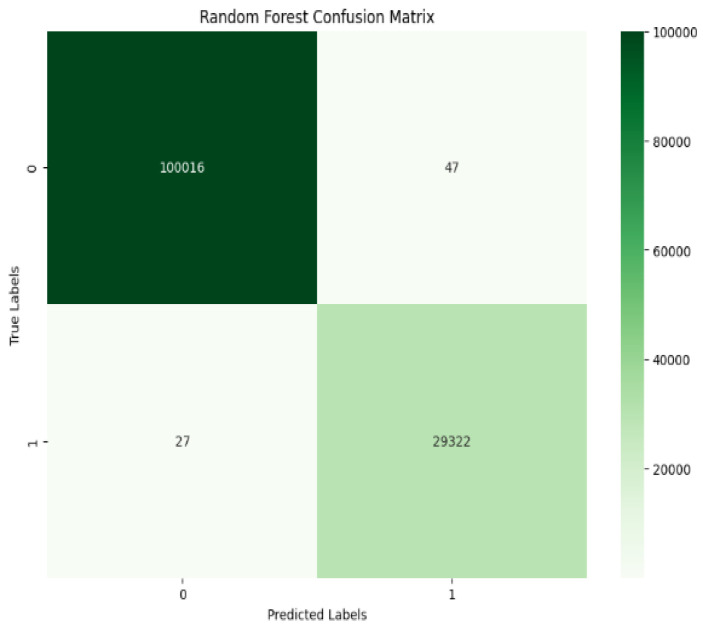
Testing confusion matrix of Random Forest.

The results demonstrate that using GridSearchCV to adjust hyperparameters dramatically improves the performance of the proposed methods, which not only improves accuracy, precision, recall, and F1-score, but also focuses on reducing computational time. RF achieves exceptional performance with 99% in all evaluation matrices shown in Table 6 and Figure 8 while showing low computational times of 6.29 s. For the Random Forest classifier, the confusion matrix of the results in Figure 9 shows that RF achieved a result of 99% when GridSearchCV determined that the best hyperparameters were n_estimators = 200, bootstrap = True, max_depth = 20, min_samples_split = 5, and min_samples_leaf = 2. The optimal hyperparameter to obtain 99.97% accuracy results is n-estimator = 200. The hyperparameter tuning configuration is presented in Table 7.

**Figure 10:**
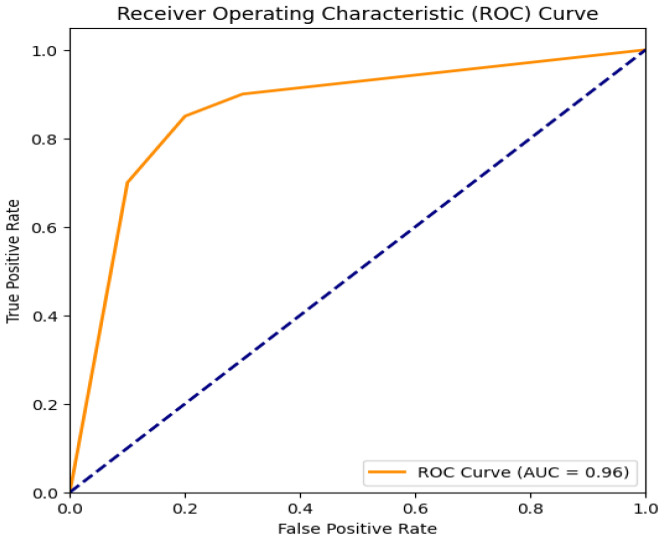
ROC curve of Random Forest.

When evaluating the performance of the classification model, the Receiver Operating Characteristic (ROC) Area Under Curve (AUC) in Figure 10 is essential. An impressive accomplishment where the model has excellent discriminating powers is representred by an AUC value of 0.96. A ROC curve that reaches the upper left corner represents the model’s capacity to accurately categorize every occurrence while ensuring a complete lack of false positives. An AUC score of this kind shows that the model is excellent at differentiating between positive and negative classes and does not incorrectly classify. The RF model’s performance is exhibited in the AUC of 0.96, a rarely seen and unusual event in practice. The model’s performance is lower than random guessing, according to values below 0.5. At the same time, an AUC of 0.5 indicates that the model performs no better than random chance. With the help of the AUC, the total discriminatory power measured provides information on the model’s classification accuracy and applicability in a range of real-world scenarios.

**Figure 11:**
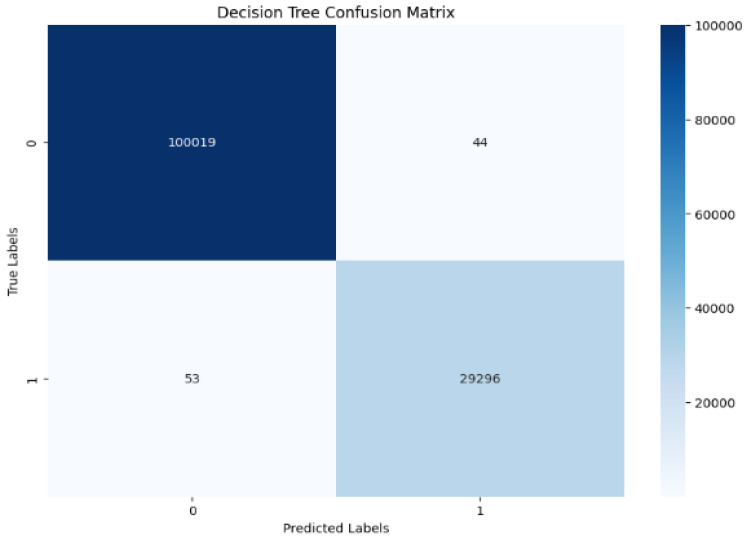
Testing confusion matrix: Decision Tree.

**Figure 12:**
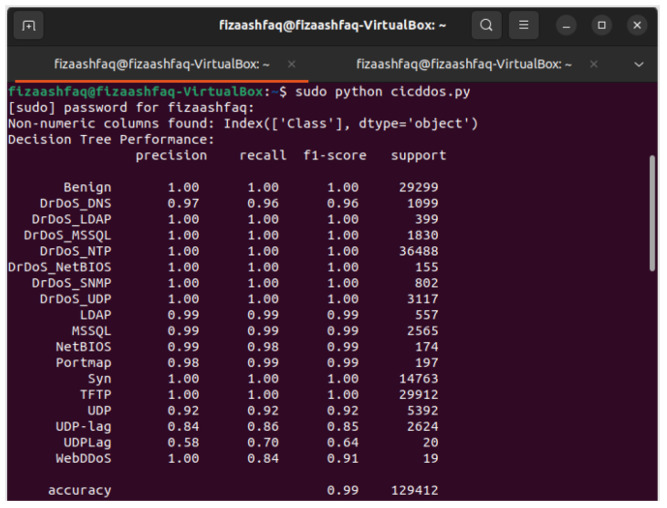
Decision Tree performance metric evaluation on the Mininet SDN network.

**Figure 13:**
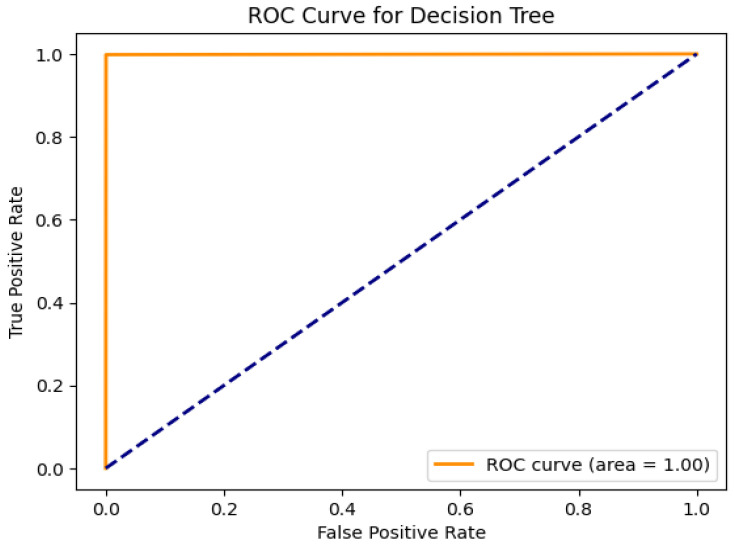
ROC curve of Decision Tree.

For the DT classifier, the confusion matrix of the results in Figure 11 shows that the DT achieves exceptional performance with 99% in all evaluation metrics as shown in Figure 12, while showing low computational times of 1 s. The perfect performance of the DT model is reflected in the AUC of 1.00 shown in Figure 13, which is a rare and unusual event in practice. KNN also maintains a high accuracy of 99%, requiring slightly more computational time at 7.3 s. SVM shows significant improvements in accuracy and F1-score, up to 99%. GridSearchCV optimizes SVM hyperparameters over C, gamma, and kernel values. To provide reliable findings, a 5-fold Stratified Cross-Validation uses ‘C’:10, ‘gamma’:‘scale’, and ‘kernel’:‘rbf’. Still, its computational time is the highest among all models at 184.5 s, which could limit its suitability for time-critical tasks.

**Figure 14:**
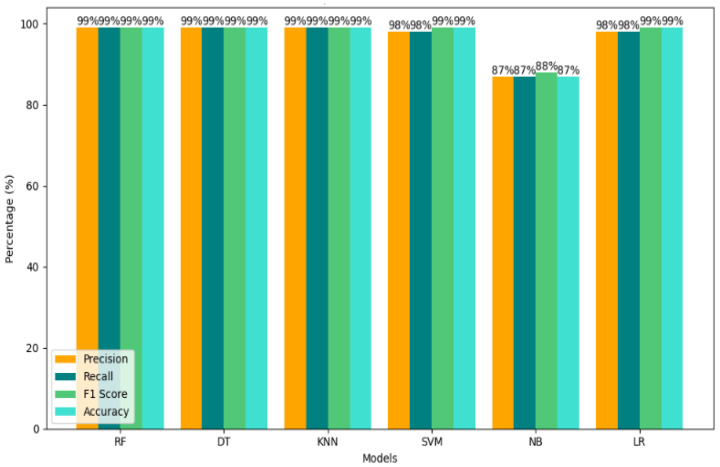
Comparison chart of machine learning mModels by performance metrics.

Naive Bayes shows improved performance with scores between 87% and 88%, but its computational time of 74.2 s is moderate. LR sustained strong results, slightly improving the computational time of 150.5 s. In general, our findings indicate that our method prioritizes both accuracy and efficiency. The comparison graph of our results is shown in Figure 14. RF and DT are particularly well suited for real-time detection due to their high accuracy and low computational time, making them ideal for efficiently mitigating DDoS attacks. A comparison of our proposed results with previous methods is given in Table 8. Previous and proposed research on attack traffic identification using machine learning methods is presented in Table 9.

**Figure 15:**
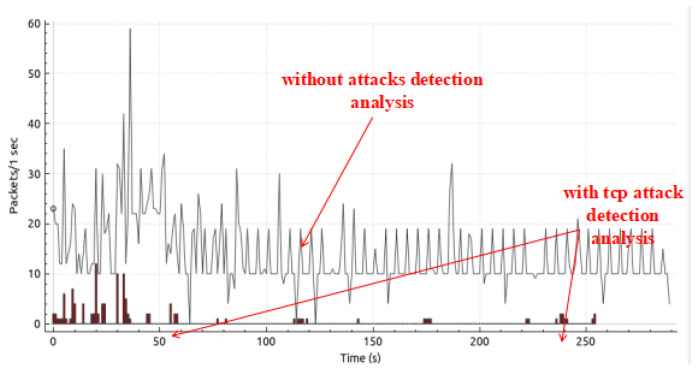
Traffic Analysis I/O graph.

**Figure 16:**
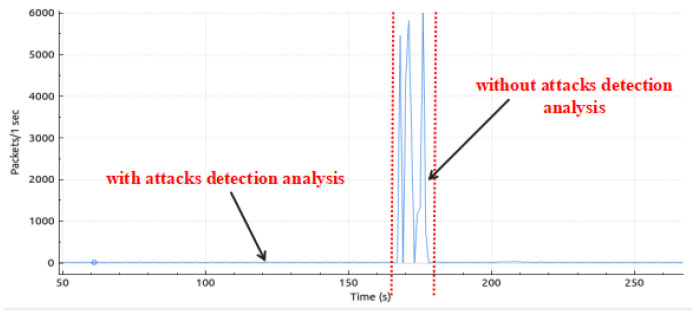
Real-time traffic analysis with and without attacks detection.

**Figure 17:**
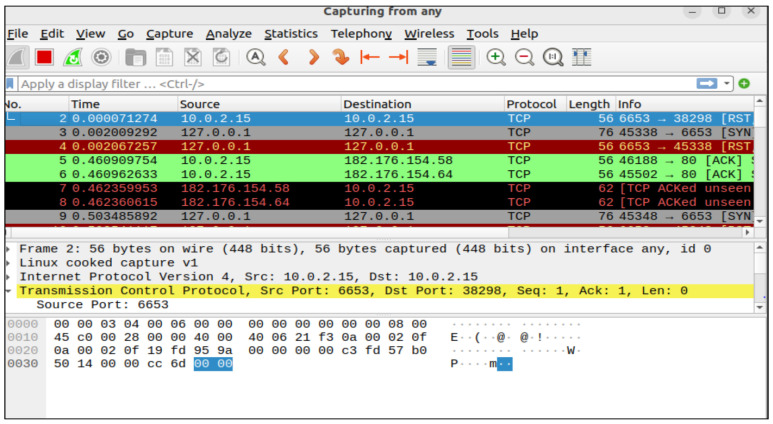
Controller-detected attack mitigation.

**Figure 18:**
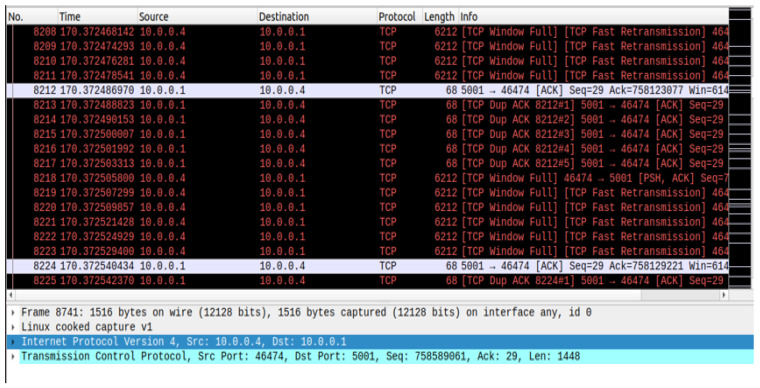
Analysis of attack detection and mitigation.

We capture traffic analysis through Wireshark. The traffic analysis I/O graph is shown in Figure 15, and the real-time traffic analysis attack detection I/O graph (with and without attacks packet flow) is presented in Figure 16. Attack detection and mitigation analyses are shown in Figure 17 and Figure 18.

## 6. Discussion

This research study addresses the problem controllers deployed in software-defined networking systems face. The primary objectives were establishing an SDN network, creating network flows that mimic attacks and mitigation, and applying a machine learning technique to detect and immediately stop these flows. In a Mininet emulation environment, we successfully implemented this method, and the above data demonstrate how well it worked to identify and mitigate SDN threats. Firewalls, intrusion prevention systems, and many more are available in conventional networking setups, but their capabilities are restricted because they rely on signatures kept in their security database. Based on Python, we developed and used a machine-learning method and mechanism for network traffic analysis using the CICDDoS2019 dataset. The results demonstrate that using GridSearchCV to adjust hyperparameters dramatically improves the performance of proposed methods, which not only improves accuracy, precision, recall, and F1-score but also focuses on reducing computational time. Random Forest and Decision Tree achieved exceptional performance with 99% across all evaluation metrics. That shows that RF and DT are particularly well suited for real-time detection because they combine high accuracy and low computational time, making them ideal for mitigating DDoS attacks effectively. The outcomes show how well machine learning-based attack mitigation strategies work to lower attack traffic and enhance network efficiency.

The controller analyzes every network transaction and produces metrics input into a machine-learning detection algorithm to determine whether an attack occurred. After capturing the network traffic through Wireshark, the controller utilizes the model detection to determine whether there is an attack and classifies it based on the network parameters. According to Amrish et al. [30], who have also used machine learning methods to prevent attacks in SDN, dynamic flow management and reconfiguration methodology significantly reduces the detection of attack traffic. Their findings show that in SDN systems, machine learning-based strategies may efficiently reduce the effects of assaults and enhance network performance. By dropping and blocking impacted ports, our mechanism shows an outstanding mitigation strategy in line with research on flexible flow monitoring and modification by Kousar et al. [47]. Our method dynamically changes the network flow rules to discard or redirect traffic associated with identified activities. By altering the network flow patterns, malicious traffic is isolated or redirected to reduce its impact on legitimate traffic. Our findings and discussion collectively demonstrate how machine learning methods, particularly Random Forest and Decision Trees, improve SDN attack detection and mitigation. This study sheds light on their functionality and how they affect network performance and offers recommendations for further research in actual business networks.

## 7. Conclusions

This paper presented a framework for designing and executing network operations by programming with software-defined networks, which is impossible with traditional networks. Although SDN is a potential future technology that is revolutionizing the networking sector, companies have only applied it in a small number of industry applications at this time. This study aimed to protect the controller by identifying and preventing any intrusion. SDN can become more intelligent, adaptable, and able to ward off complex threats by utilizing machine learning techniques. In conclusion, the methods proposed for detecting and mitigating DDoS attacks showed strong performance in accuracy, precision, recall, F1-score, and computational efficiency compared to prior studies. Models like RF and DT achieved 99% accuracy while maintaining low computational times, making them well-suited for real-time applications. KNN also performed well but required slightly more computational time. Although SVM achieved notable accuracy improvements, its high computational time limits its use in time-sensitive tasks. Naive Bayes showed moderate improvements, and LR maintained reliable performance with reduced computational times. Overall, the combination of high accuracy and computational efficiency ensures that the proposed methods are practical for real-time DDoS detection and mitigation, with RF and DT being the most balanced and valuable models. With the advancement of machine learning and software-defined networks, researchers must conduct more research and collaborate to navigate the associated challenges of this technology for network security. Although the outcomes from the CIC-DDoS2019 dataset are encouraging, it is vital to understand that the applicability of the dataset may be restricted. Examining reaction and computational time in real-time DDoS attack scenarios is equally essential and a crucial component of cybersecurity.

## Figures and Tables

**Table 1:** Analysis of supervised learning methods for intrusion detection system.

Reference	Year	Methods	Deployment and Assessment	Benefits	Accuracy (%)
[23]	2018	SVM	It performs admirably in complex fields	Intrusion detection, DDoS assaults	90
[26]	2019	SVM	SVM acquire valuable information, strong capacity	DDoS detection	94
[27]	2019	ML-Based Methods	DT handle continuous and discrete data	DoS, DDoS attacks detection using the “Long Tail” public data collection	91
[28]	2020	SVM	SVM provides precise classification for detecting DDoS attacks	Denial-of-service attacks detection	89
[29]	2023	SVM	Improves intrusion detection rate accuracy and, dangers to security	Identification of security risks	80
[3]	2024	RF, DT	When paired with DTs, supervised RF increases the classifier’s resilience	Selecting the most discriminating traits	83

**Table 2:** Comparison with previous studies (CT = Computational Time).

Reference	Year	Methods	SDN Focused	Accuracy	Simulated Environment	Sensitivity	CT
[28]	2020	KNN, SVM, NB	×	✓	✓	✓	×
[34]	2020	KNN	✓	✓	×	×	×
[12]	2021	SVM, RF, LR, NB, DT	✓	✓	✓	×	×
[29]	2023	SVM	✓	✓	×	×	×
[3]	2024	RF, DT	×	✓	✓	✓	✓
[35]	2024	SVM, DT	✓	✓	✓	✓	×
[36]	2024	DT, SVM, LR, RF	✓	✓	✓	✓	×
Proposed method	2024	DT	✓	✓	✓	✓	✓

**Table 3:** Previous studies using CICDDoS2019 dataset (RTD = Real Time Detection, SDN-C = SDN-Controller).

Ref.	Year	SDN-C	RTD	Dataset	Features/Features Selection Methods	Data Preprocessing
[45]	2021	NO	NO	CICDDoS2019	23 features by ranker search method	Remove redundant and irrelevant values, null values (binary classification)
[6]	2023	NO	NO	CICDDoS2019	20 features selected by extra tree classifier	Standard scalar normalizing, dealing with missing values, null values and transforming categorical values, label encoding (binary classification)
[3]	2024	NO	NO	CICDDoS2019	22 features selected by using Pearson’s method	Remove outliers (binary classification and multi-classification)
[46]	2024	Yes	No	CICDDoS2019	26 features selected manually and feature engineering	Standard scalar normalizing and handle redundant and missing values, label encoding (binary classification)
Our Study	2025	Yes	Yes	CICDDoS2019	20 features selected by filter-based selection	Normalizing data and handle redundant and missing values, remove outliers, transforming categorical values, label encoding (binary classification)

**Table 4:** Statistics of CICDDoS2019 dataset.

Sr.No	Class	Numbers
1	Benign	5686
2	DDoS-DNS	5,071,011
3	DDoS-LDAP	2,179,930
4	DDoS-MSSQL	4,522,492
5	DDoS-NetBIOS	4,093,279
6	DDoS-NTP	1,202,642
7	DDoS-SNMP	5,159,870
8	DDoS-SSDP	2,610,61
9	DDoS-SYN	1,582,289
10	DDoS-TFTP	20,082,580
11	DDoS-UDP	134,645
12	DDoS-UDP-Lag	366,461
13	DDoS-WebDDoS	439

**Table 5:** Requirements for the experimental environment.

Component	Description
Processor	64-bit
RAM	12 GB
Operating System	Windows 10
Virtualization Software	Oracle’s Virtual Box
Host Operating System	Ubuntu 20.04
Network Emulator	Mininet
SDN Controller	POX
Internet	An internet connection modem

**Table 6:** Results of the Random Forest tree.

Accuracy	Precision	Recall	F1-Score	Computational Time
99.97	99	**99**	99	6.29 s

**Table 7:** Hyperparameter grid search results.

Hyperparameters	Tested Values
n_estimators	50, 100, 200
max_depth	10, 20, None
min_samples_split	2, 5, 10
min_samples_leaf	1, 2, 4
bootstrap	True, False

**Table 8:** Comparison of proposed methods results and previous results. Here, Pre: Precision, Rec: Recall, F1: F1-score, Acc: Accuracy, and CT: Computational Time.

Models	Proposed Methods Results					Previous Results [12]				
	Pre (%)	Rec (%)	F1 (%)	Acc (%)	CT (s)	Pre (%)	Rec (%)	F1 (%)	Acc (%)	CT (s)
RF	99	99	99	99	6.29	98	98	99	98	84.2
DT	99	99	99	99	1	99	98	99	98	4.5
KNN	99	99	99	99	7.3	99	99	99	98	3.5
SVM	98	98	99	99	184.5	86	87	85	86	7.2
NB	87	87	88	87	74.2	66	54	38	45	1.3
LR	98	98	99	99	150.5	98	98	99	98	5.5

**Table 9:** Comparison with the outcomes of other studies.

Ref.	Models	Accuracy	Precision	Recall	F1-Score	CT
[12]	DT	98	99	98	99	4.53 s
[3]	RF	99	99	99	99	12 m
[35]	SVM	93	92	94	93	—
[28]	KNN	95	97	93	95	—
OurStudy	DT	99	99	99	99	1 s

## Data Availability

Data are contained within the article.

## Abbreviations

The following abbreviations are used in this manuscript:

ML	Machine Learning
DL	Deep Learning
RF	Random Forest
KNN	K-Nearest Neighbor
LR	Logistic Regression
SVM	Support Vector Machine
DT	Decision Tree
NB	Naive Bias
SDN	Software Defined Network
5G	5th generation
ZTA	Zero Trust Architecture
WAN	Wide Area Network
IoT	Internet Of Things
DOS	Denial of Service
DDOS	Distributed Denial of Service
U2R	User To Root
R2L	Remote to Local
IDS	Intrusion Detection System
IPS	Intrusion Prevention System
HMM	Hidden Markov Mode
DNN	Deep Neural Network
IZT	Intelligent Zero Trust
